# *MYOD-1* in normal colonic mucosa – role as a putative biomarker?

**DOI:** 10.1186/1756-0500-5-240

**Published:** 2012-07-11

**Authors:** Ramesh P Arasaradnam, M Nabil Quraishi, Daniel Commane, John C Mathers, Mike Bradburn

**Affiliations:** 1 Department of Gastroenterology, University Hospital Coventry & Warwickshire, Coventry, UK; 2 Clinical Sciences Research Institute, Warwick Medical School, Clifford Bridge Road, Coventry, CV2 2DX, UK; 3 Human Nutrition Research Institute, Newcastle University, Newcastle, UK; 4 Department of Colorectal Surgery, Wansbeck General Hospital, Wansbeck, Northumberland, UK

**Keywords:** *MYOD-1*, Colorectal cancer, Age, Promoter methylation

## Abstract

**Background:**

DNA methylation of promoter-associated CpG islands of certain genes may play a role in the development of colorectal cancer. The *MYOD-1* gene which is a muscle differentiation gene has been showed to be significantly methylated in colorectal cancer which, is an age related event. However the role of this gene in the colonic mucosa is not understood and whether methylation occurs in subjects without colon cancer. In this study, we have determined the frequency of methylation of the *MYOD-1* gene in normal colonic mucosa and investigated to see if this is associated with established colorectal cancer risk factors primarily ageing.

**Results:**

We analysed colonic mucosal biopsies in 218 normal individuals and demonstrated that in most individuals promoter hypermethylation was not quantified for *MYOD-1*. However, promoter hypermethylation increased significantly with age (p < 0.001 using regression analysis) and this was gender independent. We also showed that gene promoter methylation increased positively with an increase in waist to hip (WHR) ratio – the latter is also a known risk factor for colon cancer development.

**Conclusions:**

Our study suggests that promoter gene hypermethylation of the *MYOD-1* gene increases significantly with age in normal individuals and thus may offer potential as a putative biomarker for colorectal cancer.

## Background

Genetic alterations leading to mutations in tumour suppressor genes and oncogenes only characterise 10% of colorectal cancers. Various genetic and epigenetic modifications that lead to heritable changes in gene function but unrelated to changes in DNA sequence are understood to be mechanisms that cause development of cancer [1-4]. One of the most studied changes that are linked to neoplasia is aberrant DNA methylation. Gene specific hypermethylation can result in reduced gene expression/silencing with consequent detriment to the colonocyte, particularly if key genes such as tumour suppressor genes are involved [5,6]. It has also been suggested that detecting hypermethylation of these genes in blood or stool samples may prove to be a non invasive method of detecting those at risk of colon cancer [7].

DNA hypermethylation has been investigated extensively in colorectal cancer and other pre-malignant diseases and many genes specifically affected by CpG methylation have been identified. The *MYOD-1* gene is one such gene which is located on chromosome 11, is mainly expressed in skeletal muscle. Several studies have demonstrated hypermethylation of this gene in tumour tissue and to a lesser extent in the normal adjacent colon [8,9]. The function of this gene is of particular interest as although it is a specific muscle regulator, it is capable of inducing myogenesis in other cell types and may interact with other stem cell lineages [10,11]. However, its role in the morphologically normal colon is poorly studied as all the studies have been carried out to look at its epigenetic marking in colorectal adenomas & cancer. Ahuja et al. [8] have shown significant hypermethylation of *MYOD-1* in colon cancer tissue compared to adjacent normal mucosa and this also increases with in age.

Unfortunately there is a paucity of robust studies looking at the role of these genes in the colon and the state of methylation in healthy individuals as this would help appreciate the epigenetic changes with certain risk factors associated with colon cancer. This study proposes to look at the gene specific methylation patterns of *MYOD-1* gene within the colon in normal individuals, in an attempt to understand its relationship with known risk factors of colon cancer including age, lifestyle and anthropometric measures (waist to hip ratio (WHR)).

## Results

**Figure 1:**
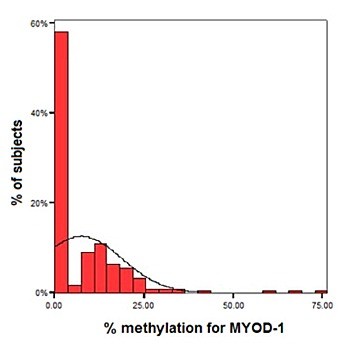
**(attached): *Percentage of methylation of MYOD-1.***

No outliers were identified for methylation of the promoter regions *MYOD-1*. Frequency histograms for the methylation data are shown in Figure 1. The observed distribution observed for *MYOD-1* was 60% of individuals with no detectable levels of methylation, 30% with methylation levels of 10 – 25% and a small proportion (< 5%) with higher levels of methylation; between 50 – 75%.

### Age and gene specific methylation pattern for *MYOD −1*

Logistic regression analysis demonstrated that methylation of *MYOD-1* was highly significantly associated with age (p < 0.001) with a greater proportion of individuals over the age of 40 having detectable methylation. No association with sex was noted after adjusting for age. As it is well recognised that the incidence of colorectal cancers doubles after the age of 40, further analysis was performed in these two groups– see Table 1. A three year follow up of these patients revealed that none of these patients developed colon cancer.

**Table 1:** **Summary of percentage methylation for*MYOD-1*and subgroup analysis of those under and over 40 years of age**

	***MYOD-1% (sd)***
Male	8.60 (14)
Female	6.10 (9.1)
Total	7.31 (11)
**Age Group**	
< 40 years	3.59 (11)
≥ 40 years	8.48 (11)

### Anthropometric measures and lifestyle factors and methylation of *MYOD −1*

WHR but not BMI was significantly (p = 0.024) positively associated with detectable *MYOD-1* gene methylation after adjusting for age. There were no associations between smoking habit, or alcohol consumption and detectable *MYOD-1* methylation.

## Discussion

This study is the largest to date which has endeavoured to characterise patterns of methylation of *MYOD-1* gene in individuals with normal colorectal mucosa. This gene has been shown previously to demonstrate an increase in methylation with age and in colorectal tumour tissue. The majority of individuals in this study had either no or low detectable methylation of *MYOD-1* which implies that in normal individuals this gene is not silenced through hypermethylation and may have a distinct role in disease whether it is age related, inflammatory or neoplastic. To date no studies have looked at the role of *MYOD-1* in colonic mucosa and whether it plays a role as a tumour suppressor.

We have demonstrated that hypermethylation on *MYOD-1* was significantly higher in subjects aged over 40. For every decade increase in age of this study population, there was a 10% increase in percentage detectable methylation [12]. This is consistent with the study performed by Ahuja et al. [8] and Hiranuma et al. [9] which showed a significant age related methylation of *MYOD-1* in normal colonic mucosa and neoplastic mucosa. Age accounted for up to 11% and WHR 13% of the proportion of variation in *MYOD-1* methylation. WHR is an important index of marker for colorectal cancers and methylation of certain genes (*HMLH1*) have been observed more frequently in those who are overweight [13].

Colorectal cancers are amongst the few cancers subjected to the intensive DNA methylation profiling, where changes in methylation in tumour samples were classified as age related as well as the cancer specific [14]. This methylation profile of multiple genes including *MYOD-1* is called “CpG island methylation phenotype (CIMP)”, and it is suggested that an unknown genetic mechanism in colorectal cancer may contribute to the clustering profile of the DNA methylation of the multiple genes together [15]. One such study by Xiao-Li Xu [16] that looked at the methylation profile of 31 genes demonstrated that altered promoter CpG island methylation patterns of 17 genes (including *MYOD-1*) were common with distinguished hallmarks specific to the early phase of colon carcinogenesis. However there was no significant correlation detected between the changes in the methylation patterns with any given clinical-pathological features apart from ageing. This adds support to findings in several studies which demonstrated that methylation of this gene increases with age and is present in colorectal cancers.

Our study reinforces the significance of methylation of certain genes that may lead to development of colorectal cancer. The role of *MYOD-1* gene in the colon is not well understood. One study showed that it played a role in the intestine smooth muscle development in ducks as the gene was highly expressed in the small intestines during the embryonic phase. Gene expression dropped rapidly following embryogenesis but it is unclear if it played an active role in adult development [17]. The significance of promoter methylation of *MYOD-1* as its function in normal colonic mucosa remains unclear.

As this was primarily an observational study there were a few limitations with it. We did not expect *MYOD-1* to be methylated in normal colonic tissue and this was a novel finding. Further studies conducted to examine the role of *MYOD-1* methylation as a biomarker should be case controlled against a colorectal cancer population in order to draw sensitivities and predictive values. Future studies should also compare *MYOD-1* with MLH-1 methylation to see how this correlates together with increasing risk of colorectal cancer. Taken together our findings lends support to the hypothesis that promoter methylation of *MYOD-1* may be an early biomarker of CRC risk since we have shown positive association with ageing and increasing WHR, both of which are established risk factors for developing colon cancer [18].

Future studies should also include measurement of mRNA levels of *MYOD-1* gene in normal colonic mucosa and specifically in colorectal cancers and correlation in mutated crypts.

## Conclusion

We have for the first time demonstrated that promoter methylation of *MYOD-1* gene is a potential biomarker for detecting risk of colorectal cancer and warrants further research to establish its role into clinical practice.

## Methods

218 subjects (105 male, 113 female) were analysed as part of the BORICC study. All patients had normal endoscopic examinations – main symptom was overt rectal bleeding. They did not have any significant past medical history and their investigations were all normal. Histological findings of colonic mucosal biopsies were also reported as normal. The baseline characteristics of these subjects are defined in Table 2. Gene specific DNA methylation was quantified in DNA from colonic mucosal biopsies and was expressed as percentage methylation of *MYOD-1*. Additional measurements made include demographic details, smoking habit and anthropometric measures.

**Table 2:** Summary of subject characteristics

**Characteristics (n = 218)**	
Male	105
Female	113
Age <40	52
Age >40	166
Mean age (sd)	50 (13)
Mean BMI (sd)	28 (6)
Mean WHR	0.89 (0.09)
Smokers	51 (23%)

### Assessment of DNA methylation

Tissue mucosal samples weighed 3–5 mg and DNA was extracted as per the manufacturer’s protocol (QIAGEN, QIAmp® DNA mini kit). Proteinase K was added and samples were heated to 56oC overnight to ensure complete tissue lysis. Additional RNAase treatment was carried out to ensure RNA free DNA samples. Only samples which had an A260/280 wavelength (Eppendorf® Biophotometer) ratio greater than 1.7 were utilised.

DNA methylation at specific gene loci was quantified by the COBRA technique (Combined Bisulphite Modified Restriction Enzyme Analysis) described by Xiong et al. [19] in which DNA was treated with sodium bisulphite followed by PCR and restriction enzyme digestion. The genomic region of interest was PCR amplified using primer sets designed specifically to contain no CpG dinucleotides. This step ensured that all templates were amplified equally regardless of the methylation status of the original genomic sequence. Following digestion with restriction enzymes separation of the digestion products using agarose gel electrophoresis, the DNA bands were visualised using Sybr green staining and the bands representing methylated and unmethylated sequences quantified using UVI software (UVI Tech Ltd.). In this study, the loci of interest were in the promoter regions of the *MYOD-1* gene.

#### Statistical analysis

SPSS v15 was used for all data analysis. For the purpose of statistical analysis, *MYOD-1* methylation was categorised binomially i.e. those samples with no detectable methylation (zero value) and those with detectable methylation (given a value of 1). Binary logistic regression was employed to determine its relationship with dependant variables (age, anthropometric measures etc.). Statistical significance was defined as p ≤ 0.05.

## Statement of ethical approval

Northumberland Local Research Ethics Committee, UK.

Ref: 04/Q0902/61.

## Authors’ contributions

RPA collected samples, drafted manuscript, performed experiments, intellectual content. NQ drafted manuscript, intellectual content. DC collected samples. MB edited manuscript drafts, intellectual content. JCM edited manuscript drafts, intellectual content. All authors read and approved the final manuscript.

## Competing interests

The authors declare that they have no competing interest.
